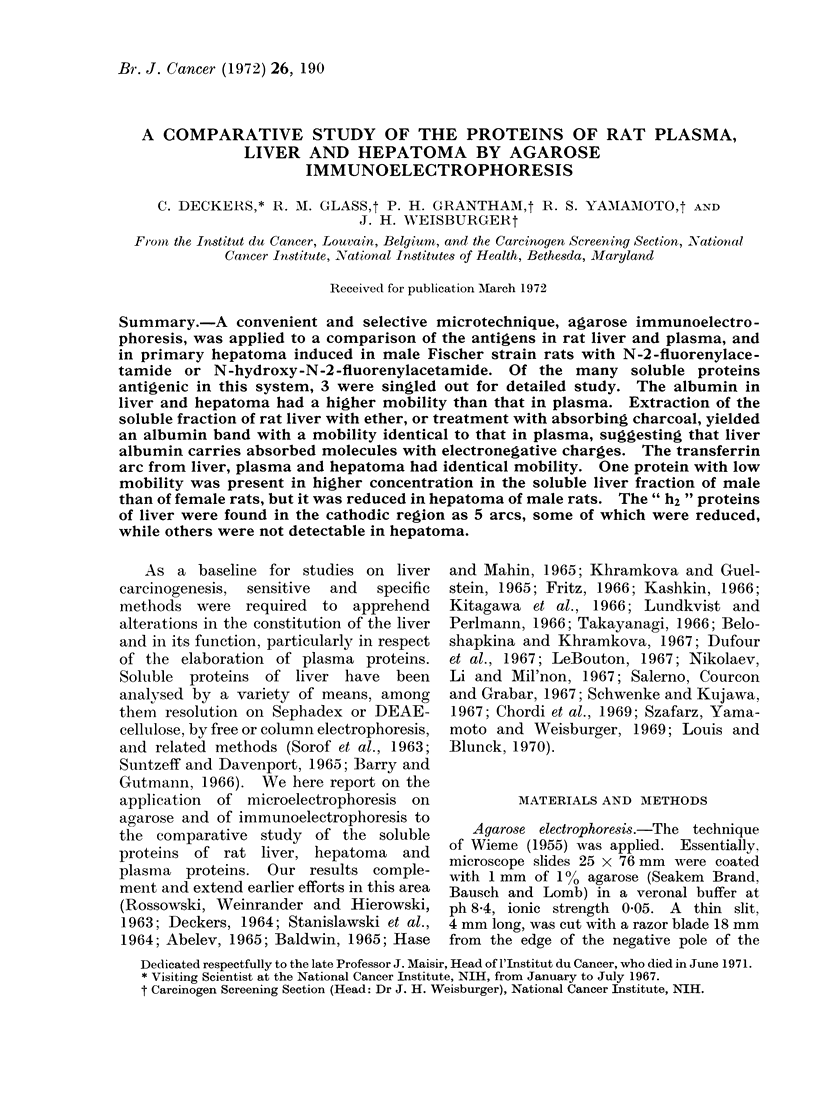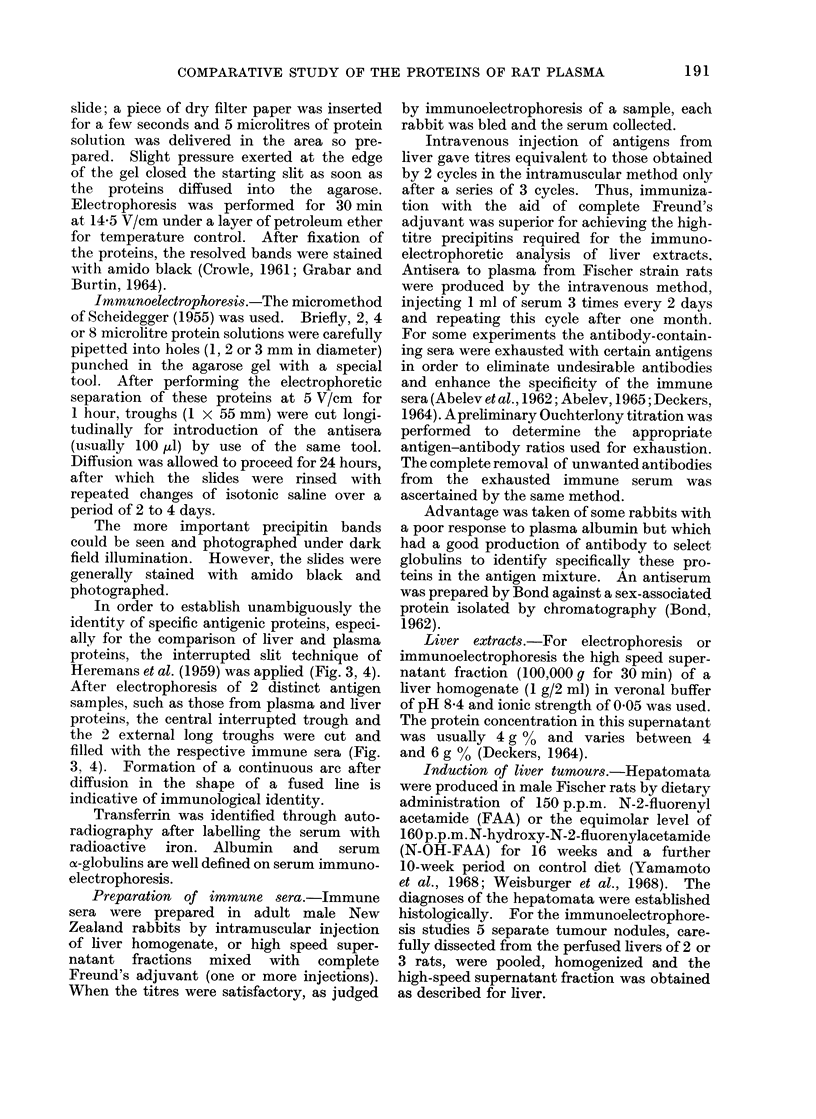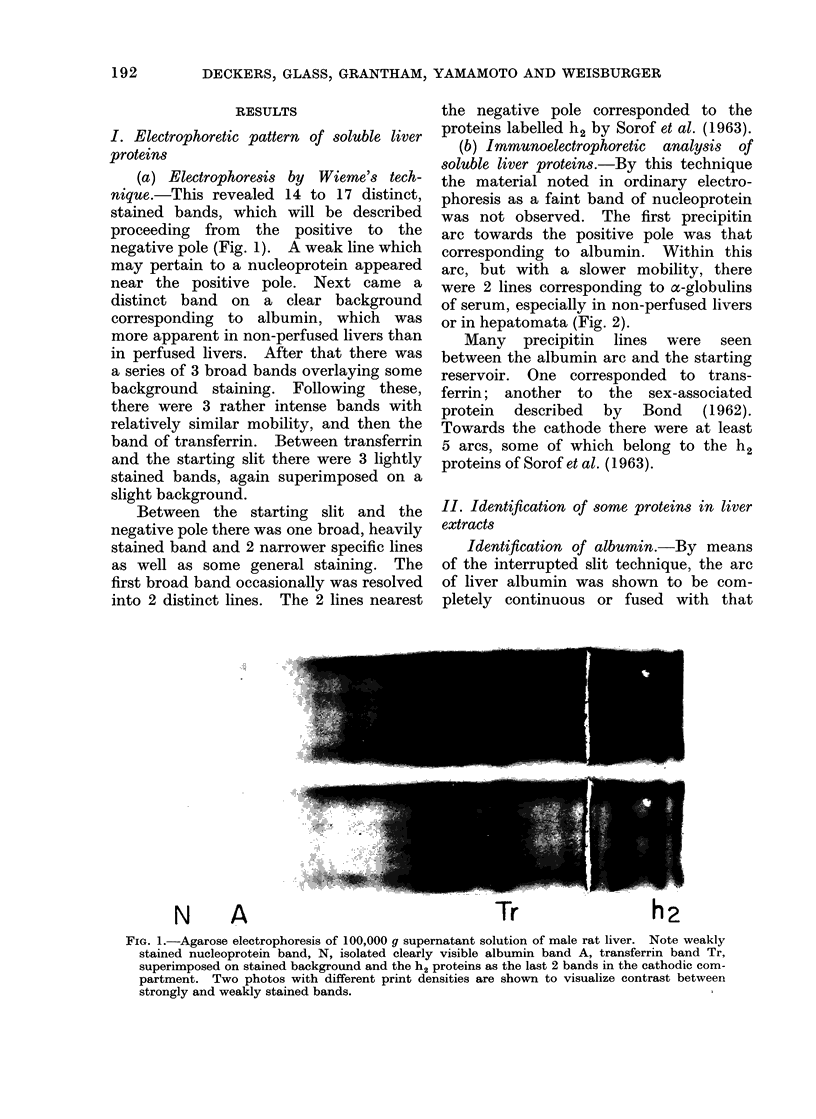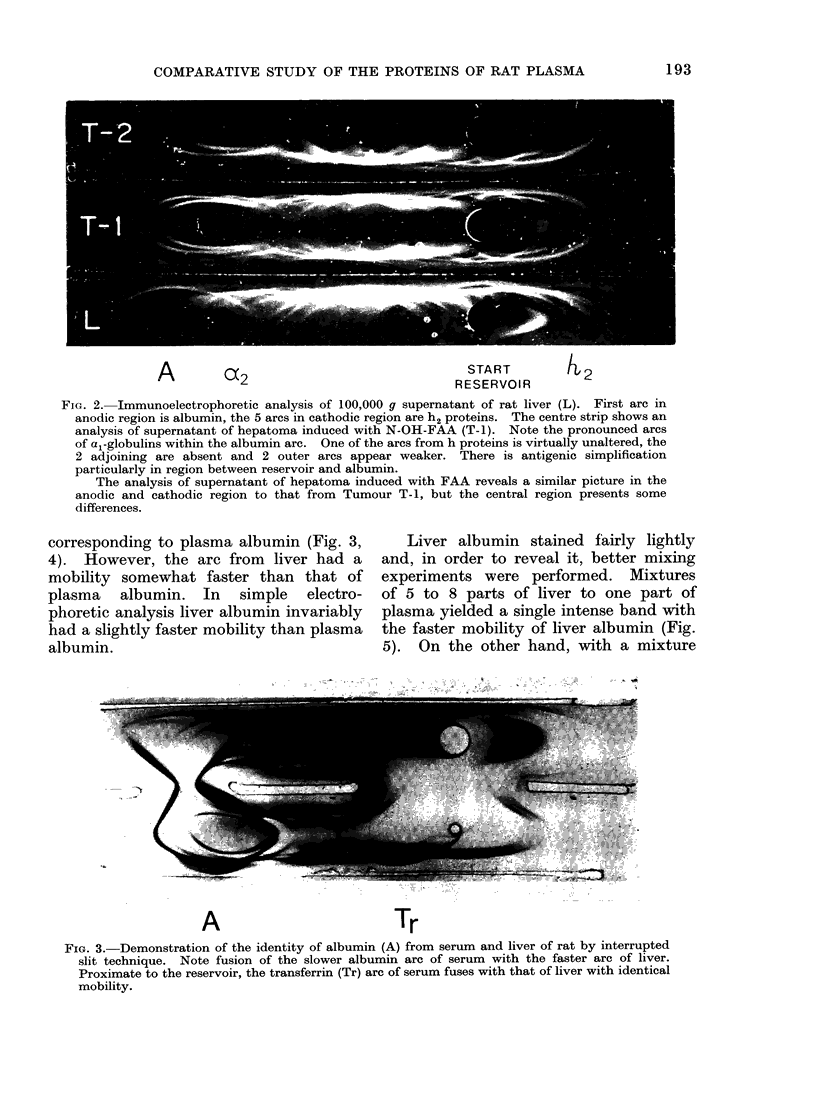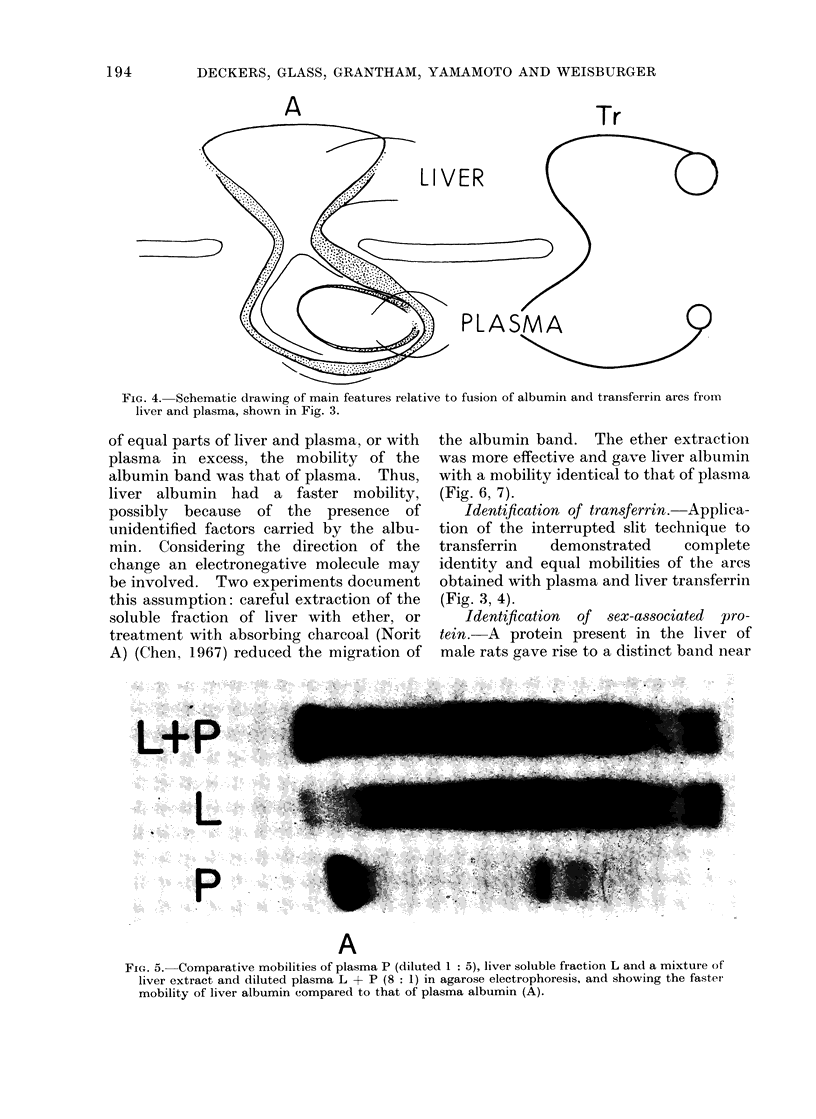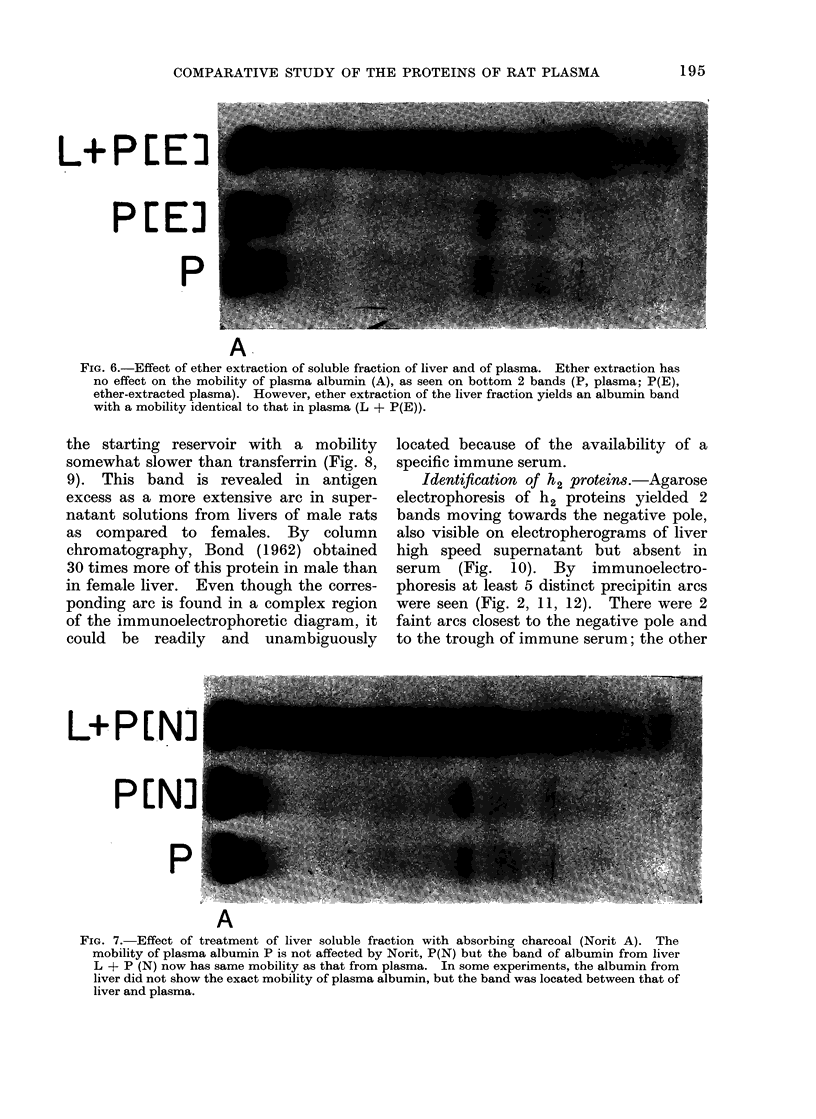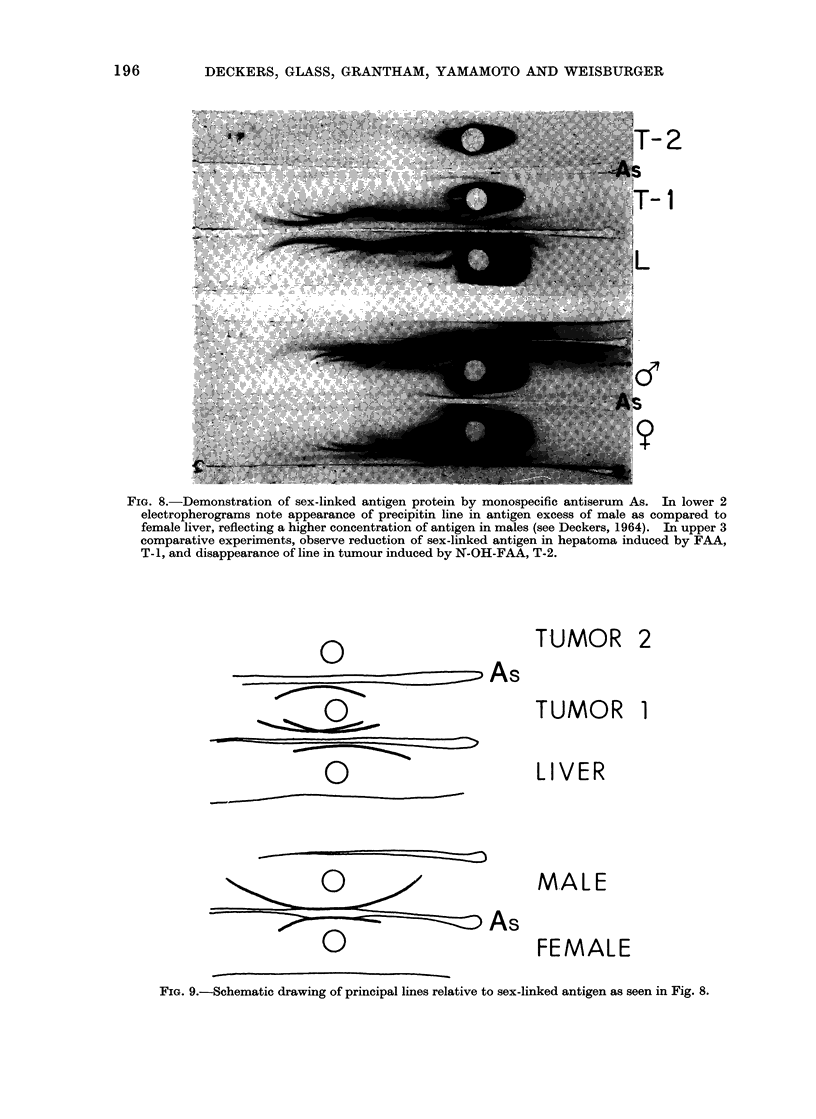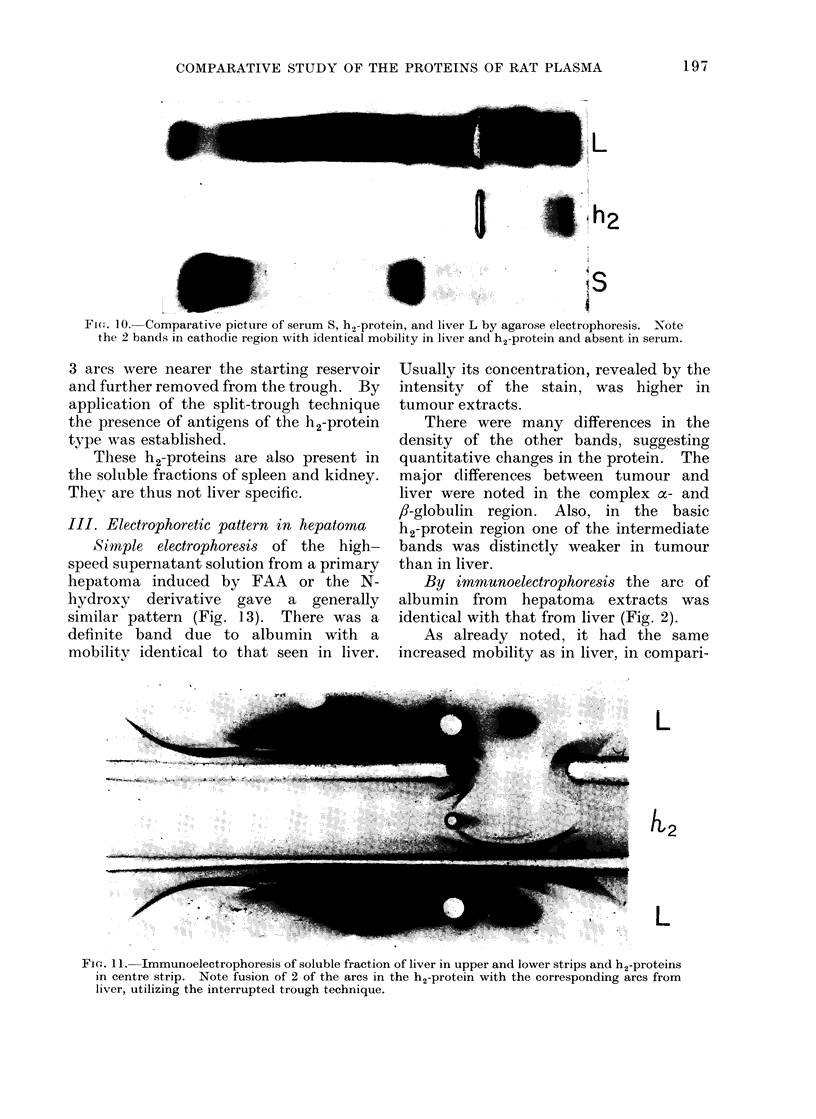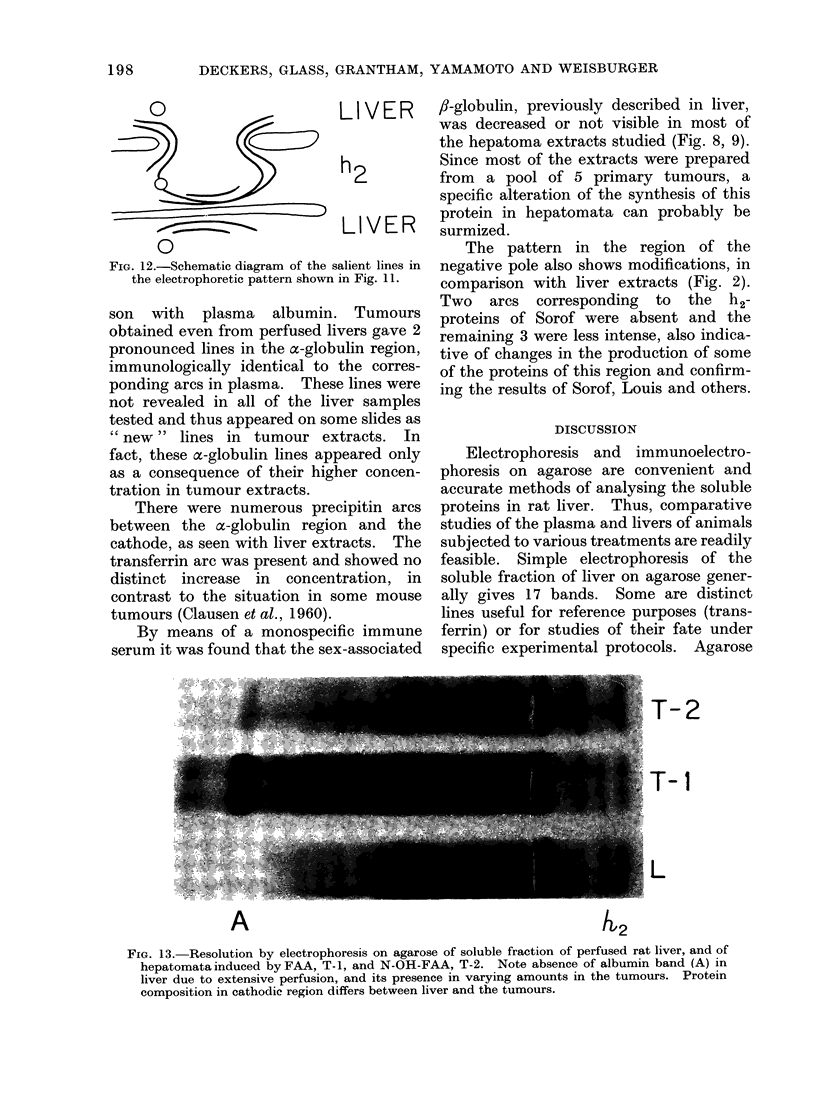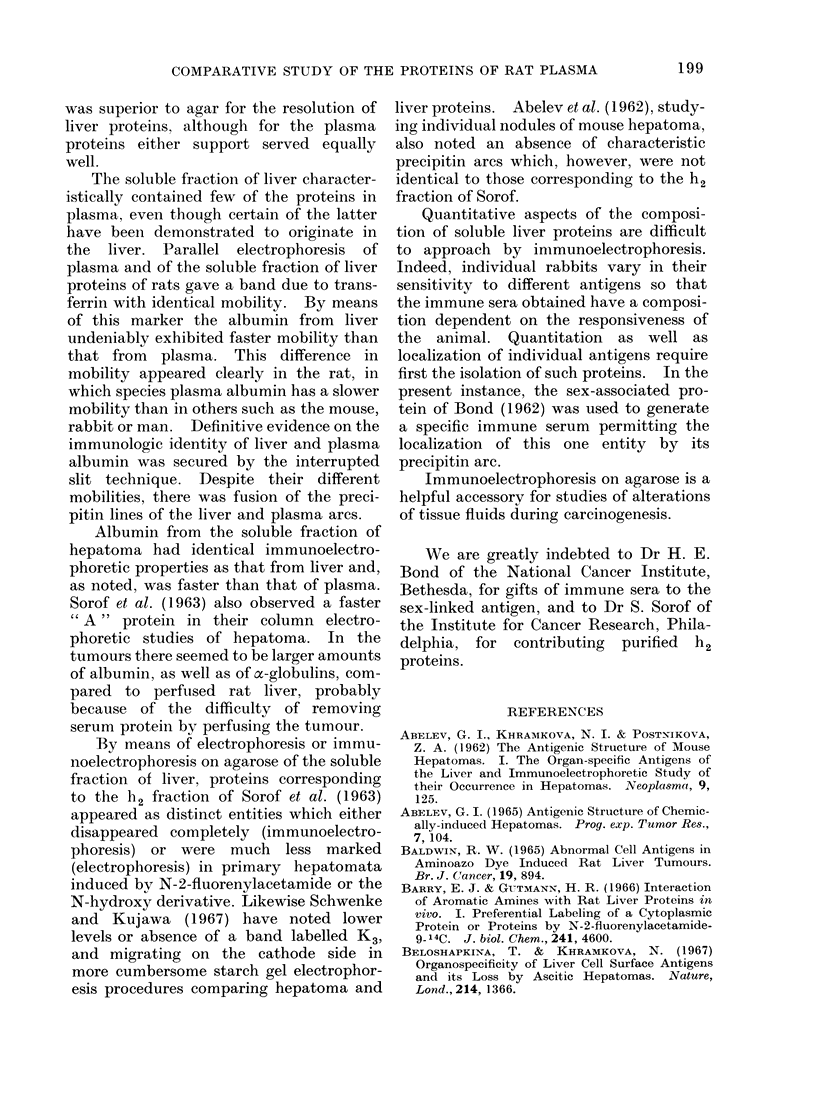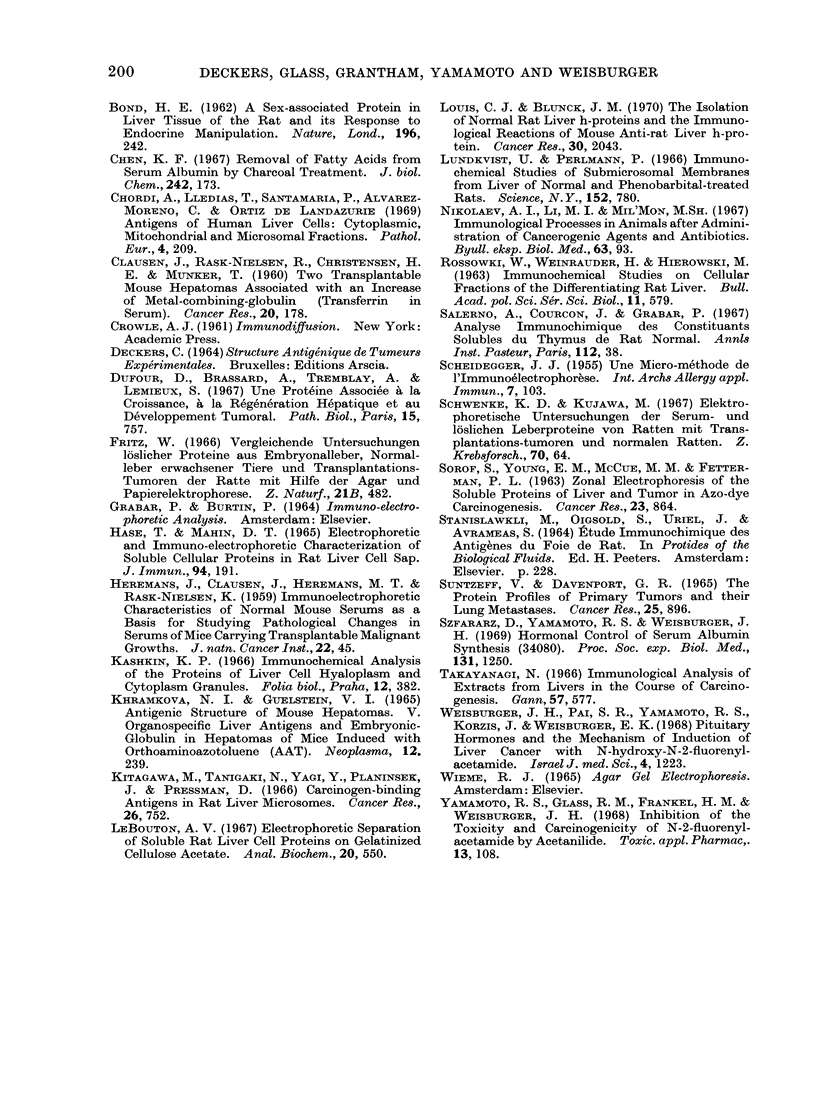# A Comparative Study of the Proteins of Rat Plasma, Liver and Hepatoma by Agarose Immunoelectrophoresis

**DOI:** 10.1038/bjc.1972.26

**Published:** 1972-06

**Authors:** C. Deckers, R. M. Glass, P. H. Grantham, R. S. Yamamoto, J. H. Weisburger

## Abstract

**Images:**


*Br. J. Cancer* (1972) **26**, 190

### A COMPARATIVE STUDY OF THE PROTEINS OF RAT PLASMA, LIVER AND HEPATOMA BY AGAROSE IMMUNOELECTROPHORESIS

C. DECKERS,* R. M. GLASS,† P. H. GRANTHAM,† R. S. YAMAMOTO,† AND J. H. WEISBURGER†

*From the Institut du Cancer, Louvain, Belgium, and the Carcinogen Screening Section, National Cancer Institute, National Institutes of Health, Bethesda, Maryland*

Received for publication March 1972

**Summary.—A convenient and selective microtechnique, agarose immunoelectrophoresis, was applied to a comparison of the antigens in rat liver and plasma, and in primary hepatoma induced in male Fischer strain rats with N-2-fluorenylacetamide or N-hydroxy-N-2-fluorenylacetamide. Of the many soluble proteins antigenic in this system, 3 were singled out for detailed study. The albumin in liver and hepatoma had a higher mobility than that in plasma. Extraction of the soluble fraction of rat liver with ether, or treatment with absorbing charcoal, yielded an albumin band with a mobility identical to that in plasma, suggesting that liver albumin carries absorbed molecules with electronegative charges. The transferrin arc from liver, plasma and hepatoma had identical mobility. One protein with low mobility was present in higher concentration in the soluble liver fraction of male than of female rats, but it was reduced in hepatoma of male rats. The " $h_2$ " proteins of liver were found in the cathodic region as 5 arcs, some of which were reduced, while others were not detectable in hepatoma.**

As a baseline for studies on liver carcinogenesis, sensitive and specific methods were required to apprehend alterations in the constitution of the liver and in its function, particularly in respect of the elaboration of plasma proteins. Soluble proteins of liver have been analysed by a variety of means, among them resolution on Sephadex or DEAE-cellulose, by free or column electrophoresis, and related methods (Sorof *et al.*, 1963; Suntzeff and Davenport, 1965; Barry and Gutmann, 1966). We here report on the application of microelectrophoresis on agarose and of immunoelectrophoresis to the comparative study of the soluble proteins of rat liver, hepatoma and plasma proteins. Our results complement and extend earlier efforts in this area (Rossowski, Weinrander and Hierowski, 1963; Deckers, 1964; Stanislawski *et al.*, 1964; Abelev, 1965; Baldwin, 1965; Hase and Mahin, 1965; Khramkova and Guelstein, 1965; Fritz, 1966; Kashkin, 1966; Kitagawa *et al.*, 1966; Lundkvist and Perlmann, 1966; Takayanagi, 1966; Beloshapkina and Khramkova, 1967; Dufour *et al.*, 1967; LeBouton, 1967; Nikolaev, Li and Mil'non, 1967; Salerno, Courcon and Grabar, 1967; Schwenke and Kujawa, 1967; Chordi *et al.*, 1969; Szafarz, Yamamoto and Weisburger, 1969; Louis and Blunck, 1970).

### MATERIALS AND METHODS

*Agarose electrophoresis.*—The technique of Wieme (1955) was applied. Essentially, microscope slides 25 × 76 mm were coated with 1 mm of 1% agarose (Seakem Brand, Bausch and Lomb) in a veronal buffer at ph 8·4, ionic strength 0·05. A thin slit, 4 mm long, was cut with a razor blade 18 mm from the edge of the negative pole of the

Dedicated respectfully to the late Professor J. Maisir, Head of l'Institut du Cancer, who died in June 1971.

* Visiting Scientist at the National Cancer Institute, NIH, from January to July 1967.

† Carcinogen Screening Section (Head: Dr J. H. Weisburger), National Cancer Institute, NIH.

slide; a piece of dry filter paper was inserted for a few seconds and 5 microlitres of protein solution was delivered in the area so prepared. Slight pressure exerted at the edge of the gel closed the starting slit as soon as the proteins diffused into the agarose. Electrophoresis was performed for 30 min at 14·5 V/cm under a layer of petroleum ether for temperature control. After fixation of the proteins, the resolved bands were stained with amido black (Crowle, 1961; Grabar and Burtin, 1964).

*Immunoelectrophoresis.*—The micromethod of Scheidegger (1955) was used. Briefly, 2, 4 or 8 microlitre protein solutions were carefully pipetted into holes (1, 2 or 3 mm in diameter) punched in the agarose gel with a special tool. After performing the electrophoretic separation of these proteins at 5 V/cm for 1 hour, troughs (1 $\times$ 55 mm) were cut longitudinally for introduction of the antisera (usually 100 $\mu$l) by use of the same tool. Diffusion was allowed to proceed for 24 hours, after which the slides were rinsed with repeated changes of isotonic saline over a period of 2 to 4 days.

The more important precipitin bands could be seen and photographed under dark field illumination. However, the slides were generally stained with amido black and photographed.

In order to establish unambiguously the identity of specific antigenic proteins, especially for the comparison of liver and plasma proteins, the interrupted slit technique of Heremans *et al.* (1959) was applied (Fig. 3, 4). After electrophoresis of 2 distinct antigen samples, such as those from plasma and liver proteins, the central interrupted trough and the 2 external long troughs were cut and filled with the respective immune sera (Fig. 3, 4). Formation of a continuous arc after diffusion in the shape of a fused line is indicative of immunological identity.

Transferrin was identified through autoradiography after labelling the serum with radioactive iron. Albumin and serum $\alpha$-globulins are well defined on serum immunoelectrophoresis.

*Preparation of immune sera.*—Immune sera were prepared in adult male New Zealand rabbits by intramuscular injection of liver homogenate, or high speed supernatant fractions mixed with complete Freund's adjuvant (one or more injections). When the titres were satisfactory, as judged by immunoelectrophoresis of a sample, each rabbit was bled and the serum collected.

Intravenous injection of antigens from liver gave titres equivalent to those obtained by 2 cycles in the intramuscular method only after a series of 3 cycles. Thus, immunization with the aid of complete Freund's adjuvant was superior for achieving the high-titre precipitins required for the immunoelectrophoretic analysis of liver extracts. Antisera to plasma from Fischer strain rats were produced by the intravenous method, injecting 1 ml of serum 3 times every 2 days and repeating this cycle after one month. For some experiments the antibody-containing sera were exhausted with certain antigens in order to eliminate undesirable antibodies and enhance the specificity of the immune sera (Abelev *et al.*, 1962; Abelev, 1965; Deckers, 1964). A preliminary Ouchterlony titration was performed to determine the appropriate antigen–antibody ratios used for exhaustion. The complete removal of unwanted antibodies from the exhausted immune serum was ascertained by the same method.

Advantage was taken of some rabbits with a poor response to plasma albumin but which had a good production of antibody to select globulins to identify specifically these proteins in the antigen mixture. An antiserum was prepared by Bond against a sex-associated protein isolated by chromatography (Bond, 1962).

*Liver extracts.*—For electrophoresis or immunoelectrophoresis the high speed supernatant fraction (100,000 *g* for 30 min) of a liver homogenate (1 g/2 ml) in veronal buffer of pH 8·4 and ionic strength of 0·05 was used. The protein concentration in this supernatant was usually 4 g % and varies between 4 and 6 g % (Deckers, 1964).

*Induction of liver tumours.*—Hepatomata were produced in male Fischer rats by dietary administration of 150 p.p.m. N-2-fluorenyl acetamide (FAA) or the equimolar level of 160 p.p.m. N-hydroxy-N-2-fluorenylacetamide (N-OH-FAA) for 16 weeks and a further 10-week period on control diet (Yamamoto *et al.*, 1968; Weisburger *et al.*, 1968). The diagnoses of the hepatomata were established histologically. For the immunoelectrophoresis studies 5 separate tumour nodules, carefully dissected from the perfused livers of 2 or 3 rats, were pooled, homogenized and the high-speed supernatant fraction was obtained as described for liver.

### RESULTS

### *I. Electrophoretic pattern of soluble liver proteins*

(*a*) *Electrophoresis by Wieme's technique.*—This revealed 14 to 17 distinct, stained bands, which will be described proceeding from the positive to the negative pole (Fig. 1). A weak line which may pertain to a nucleoprotein appeared near the positive pole. Next came a distinct band on a clear background corresponding to albumin, which was more apparent in non-perfused livers than in perfused livers. After that there was a series of 3 broad bands overlaying some background staining. Following these, there were 3 rather intense bands with relatively similar mobility, and then the band of transferrin. Between transferrin and the starting slit there were 3 lightly stained bands, again superimposed on a slight background.

Between the starting slit and the negative pole there was one broad, heavily stained band and 2 narrower specific lines as well as some general staining. The first broad band occasionally was resolved into 2 distinct lines. The 2 lines nearest the negative pole corresponded to the proteins labelled $h_2$ by Sorof *et al.* (1963).

(*b*) *Immunoelectrophoretic analysis of soluble liver proteins.*—By this technique the material noted in ordinary electrophoresis as a faint band of nucleoprotein was not observed. The first precipitin arc towards the positive pole was that corresponding to albumin. Within this arc, but with a slower mobility, there were 2 lines corresponding to $\alpha$-globulins of serum, especially in non-perfused livers or in hepatomata (Fig. 2).

Many precipitin lines were seen between the albumin arc and the starting reservoir. One corresponded to transferrin; another to the sex-associated protein described by Bond (1962). Towards the cathode there were at least 5 arcs, some of which belong to the $h_2$ proteins of Sorof *et al.* (1963).

### *II. Identification of some proteins in liver extracts*

*Identification of albumin.*—By means of the interrupted slit technique, the arc of liver albumin was shown to be completely continuous or fused with that

FIG. 1.—Agarose electrophoresis of 100,000 *g* supernatant solution of male rat liver. Note weakly stained nucleoprotein band, N, isolated clearly visible albumin band A, transferrin band Tr, superimposed on stained background and the $h_2$ proteins as the last 2 bands in the cathodic compartment. Two photos with different print densities are shown to visualize contrast between strongly and weakly stained bands.

Fig. 2.—Immunoelectrophoretic analysis of 100,000 *g* supernatant of rat liver (L). First arc in anodic region is albumin, the 5 arcs in cathodic region are $h_2$ proteins. The centre strip shows an analysis of supernatant of hepatoma induced with N-OH-FAA (T-1). Note the pronounced arcs of $\alpha_1$-globulins within the albumin arc. One of the arcs from h proteins is virtually unaltered, the 2 adjoining are absent and 2 outer arcs appear weaker. There is antigenic simplification particularly in region between reservoir and albumin.

The analysis of supernatant of hepatoma induced with FAA reveals a similar picture in the anodic and cathodic region to that from Tumour T-1, but the central region presents some differences.

corresponding to plasma albumin (Fig. 3, 4). However, the arc from liver had a mobility somewhat faster than that of plasma albumin. In simple electrophoretic analysis liver albumin invariably had a slightly faster mobility than plasma albumin.

Liver albumin stained fairly lightly and, in order to reveal it, better mixing experiments were performed. Mixtures of 5 to 8 parts of liver to one part of plasma yielded a single intense band with the faster mobility of liver albumin (Fig. 5). On the other hand, with a mixture

Fig. 3.—Demonstration of the identity of albumin (A) from serum and liver of rat by interrupted slit technique. Note fusion of the slower albumin arc of serum with the faster arc of liver. Proximate to the reservoir, the transferrin (Tr) arc of serum fuses with that of liver with identical mobility.

FIG. 4.—Schematic drawing of main features relative to fusion of albumin and transferrin arcs from liver and plasma, shown in Fig. 3.

of equal parts of liver and plasma, or with plasma in excess, the mobility of the albumin band was that of plasma. Thus, liver albumin had a faster mobility, possibly because of the presence of unidentified factors carried by the albumin. Considering the direction of the change an electronegative molecule may be involved. Two experiments document this assumption: careful extraction of the soluble fraction of liver with ether, or treatment with absorbing charcoal (Norit A) (Chen, 1967) reduced the migration of the albumin band. The ether extraction was more effective and gave liver albumin with a mobility identical to that of plasma (Fig. 6, 7).

*Identification of transferrin.*—Application of the interrupted slit technique to transferrin demonstrated complete identity and equal mobilities of the arcs obtained with plasma and liver transferrin (Fig. 3, 4).

*Identification of sex-associated protein.*—A protein present in the liver of male rats gave rise to a distinct band near

FIG. 5.—Comparative mobilities of plasma P (diluted 1 : 5), liver soluble fraction L and a mixture of liver extract and diluted plasma L + P (8 : 1) in agarose electrophoresis, and showing the faster mobility of liver albumin compared to that of plasma albumin (A).

FIG. 6.—Effect of ether extraction of soluble fraction of liver and of plasma. Ether extraction has no effect on the mobility of plasma albumin (A), as seen on bottom 2 bands (P, plasma; P(E), ether-extracted plasma). However, ether extraction of the liver fraction yields an albumin band with a mobility identical to that in plasma (L + P(E)).

the starting reservoir with a mobility somewhat slower than transferrin (Fig. 8, 9). This band is revealed in antigen excess as a more extensive arc in supernatant solutions from livers of male rats as compared to females. By column chromatography, Bond (1962) obtained 30 times more of this protein in male than in female liver. Even though the corresponding arc is found in a complex region of the immunoelectrophoretic diagram, it could be readily and unambiguously located because of the availability of a specific immune serum.

*Identification of $h_2$ proteins.*—Agarose electrophoresis of $h_2$ proteins yielded 2 bands moving towards the negative pole, also visible on electropherograms of liver high speed supernatant but absent in serum (Fig. 10). By immunoelectrophoresis at least 5 distinct precipitin arcs were seen (Fig. 2, 11, 12). There were 2 faint arcs closest to the negative pole and to the trough of immune serum; the other

FIG. 7.—Effect of treatment of liver soluble fraction with absorbing charcoal (Norit A). The mobility of plasma albumin P is not affected by Norit, P(N) but the band of albumin from liver L + P (N) now has same mobility as that from plasma. In some experiments, the albumin from liver did not show the exact mobility of plasma albumin, but the band was located between that of liver and plasma.

FIG. 8.—Demonstration of sex-linked antigen protein by monospecific antiserum As. In lower 2 electropherograms note appearance of precipitin line in antigen excess of male as compared to female liver, reflecting a higher concentration of antigen in males (see Deckers, 1964). In upper 3 comparative experiments, observe reduction of sex-linked antigen in hepatoma induced by FAA, T-1, and disappearance of line in tumour induced by N-OH-FAA, T-2.

FIG. 9.—Schematic drawing of principal lines relative to sex-linked antigen as seen in Fig. 8.

FIG. 10.—Comparative picture of serum S, $h_2$-protein, and liver L by agarose electrophoresis. Note the 2 bands in cathodic region with identical mobility in liver and $h_2$-protein and absent in serum.

3 arcs were nearer the starting reservoir and further removed from the trough. By application of the split-trough technique the presence of antigens of the $h_2$-protein type was established.

These $h_2$-proteins are also present in the soluble fractions of spleen and kidney. They are thus not liver specific.

### *III. Electrophoretic pattern in hepatoma*

*Simple electrophoresis* of the high-speed supernatant solution from a primary hepatoma induced by FAA or the N-hydroxy derivative gave a generally similar pattern (Fig. 13). There was a definite band due to albumin with a mobility identical to that seen in liver. Usually its concentration, revealed by the intensity of the stain, was higher in tumour extracts.

There were many differences in the density of the other bands, suggesting quantitative changes in the protein. The major differences between tumour and liver were noted in the complex $\alpha$- and $\beta$-globulin region. Also, in the basic $h_2$-protein region one of the intermediate bands was distinctly weaker in tumour than in liver.

*By immunoelectrophoresis* the arc of albumin from hepatoma extracts was identical with that from liver (Fig. 2).

As already noted, it had the same increased mobility as in liver, in compari-

FIG. 11.—Immunoelectrophoresis of soluble fraction of liver in upper and lower strips and $h_2$-proteins in centre strip. Note fusion of 2 of the arcs in the $h_2$-protein with the corresponding arcs from liver, utilizing the interrupted trough technique.

FIG. 12.—Schematic diagram of the salient lines in the electrophoretic pattern shown in Fig. 11.

son with plasma albumin. Tumours obtained even from perfused livers gave 2 pronounced lines in the α-globulin region, immunologically identical to the corresponding arcs in plasma. These lines were not revealed in all of the liver samples tested and thus appeared on some slides as " new " lines in tumour extracts. In fact, these α-globulin lines appeared only as a consequence of their higher concentration in tumour extracts.

There were numerous precipitin arcs between the α-globulin region and the cathode, as seen with liver extracts. The transferrin arc was present and showed no distinct increase in concentration, in contrast to the situation in some mouse tumours (Clausen *et al.*, 1960).

By means of a monospecific immune serum it was found that the sex-associated β-globulin, previously described in liver, was decreased or not visible in most of the hepatoma extracts studied (Fig. 8, 9). Since most of the extracts were prepared from a pool of 5 primary tumours, a specific alteration of the synthesis of this protein in hepatomata can probably be surmized.

The pattern in the region of the negative pole also shows modifications, in comparison with liver extracts (Fig. 2). Two arcs corresponding to the $h_2$-proteins of Sorof were absent and the remaining 3 were less intense, also indicative of changes in the production of some of the proteins of this region and confirming the results of Sorof, Louis and others.

### DISCUSSION

Electrophoresis and immunoelectrophoresis on agarose are convenient and accurate methods of analysing the soluble proteins in rat liver. Thus, comparative studies of the plasma and livers of animals subjected to various treatments are readily feasible. Simple electrophoresis of the soluble fraction of liver on agarose generally gives 17 bands. Some are distinct lines useful for reference purposes (transferrin) or for studies of their fate under specific experimental protocols. Agarose

FIG. 13.—Resolution by electrophoresis on agarose of soluble fraction of perfused rat liver, and of hepatomata induced by FAA, T-1, and N-OH-FAA, T-2. Note absence of albumin band (A) in liver due to extensive perfusion, and its presence in varying amounts in the tumours. Protein composition in cathodic region differs between liver and the tumours.

was superior to agar for the resolution of liver proteins, although for the plasma proteins either support served equally well.

The soluble fraction of liver characteristically contained few of the proteins in plasma, even though certain of the latter have been demonstrated to originate in the liver. Parallel electrophoresis of plasma and of the soluble fraction of liver proteins of rats gave a band due to transferrin with identical mobility. By means of this marker the albumin from liver undeniably exhibited faster mobility than that from plasma. This difference in mobility appeared clearly in the rat, in which species plasma albumin has a slower mobility than in others such as the mouse, rabbit or man. Definitive evidence on the immunologic identity of liver and plasma albumin was secured by the interrupted slit technique. Despite their different mobilities, there was fusion of the precipitin lines of the liver and plasma arcs.

Albumin from the soluble fraction of hepatoma had identical immunoelectrophoretic properties as that from liver and, as noted, was faster than that of plasma. Sorof *et al.* (1963) also observed a faster " A " protein in their column electrophoretic studies of hepatoma. In the tumours there seemed to be larger amounts of albumin, as well as of $\alpha$-globulins, compared to perfused rat liver, probably because of the difficulty of removing serum protein by perfusing the tumour.

By means of electrophoresis or immunoelectrophoresis on agarose of the soluble fraction of liver, proteins corresponding to the $h_2$ fraction of Sorof *et al.* (1963) appeared as distinct entities which either disappeared completely (immunoelectrophoresis) or were much less marked (electrophoresis) in primary hepatomata induced by N-2-fluorenylacetamide or the N-hydroxy derivative. Likewise Schwenke and Kujawa (1967) have noted lower levels or absence of a band labelled $K_3$, and migrating on the cathode side in more cumbersome starch gel electrophoresis procedures comparing hepatoma and liver proteins. Abelev *et al.* (1962), studying individual nodules of mouse hepatoma, also noted an absence of characteristic precipitin arcs which, however, were not identical to those corresponding to the $h_2$ fraction of Sorof.

Quantitative aspects of the composition of soluble liver proteins are difficult to approach by immunoelectrophoresis. Indeed, individual rabbits vary in their sensitivity to different antigens so that the immune sera obtained have a composition dependent on the responsiveness of the animal. Quantitation as well as localization of individual antigens require first the isolation of such proteins. In the present instance, the sex-associated protein of Bond (1962) was used to generate a specific immune serum permitting the localization of this one entity by its precipitin arc.

Immunoelectrophoresis on agarose is a helpful accessory for studies of alterations of tissue fluids during carcinogenesis.

We are greatly indebted to Dr H. E. Bond of the National Cancer Institute, Bethesda, for gifts of immune sera to the sex-linked antigen, and to Dr S. Sorof of the Institute for Cancer Research, Philadelphia, for contributing purified $h_2$ proteins.

### REFERENCES

ABELEV, G. I., KHRAMKOVA, N. I. & POSTNIKOVA, Z. A. (1962) The Antigenic Structure of Mouse Hepatomas. I. The Organ-specific Antigens of the Liver and Immunoelectrophoretic Study of their Occurrence in Hepatomas. *Neoplasma*, **9**, 125.

ABELEV, G. I. (1965) Antigenic Structure of Chemically-induced Hepatomas. *Prog. exp. Tumor Res.*, **7**, 104.

BALDWIN, R. W. (1965) Abnormal Cell Antigens in Aminoazo Dye Induced Rat Liver Tumours. *Br. J. Cancer*, **19**, 894.

BARRY, E. J. & GUTMANN, H. R. (1966) Interaction of Aromatic Amines with Rat Liver Proteins *in vivo*. I. Preferential Labeling of a Cytoplasmic Protein or Proteins by N-2-fluorenylacetamide-9-$^{14}$C. *J. biol. Chem.*, **241**, 4600.

BELOSHAPKINA, T. & KHRAMKOVA, N. (1967) Organospecificity of Liver Cell Surface Antigens and its Loss by Ascitic Hepatomas. *Nature, Lond.*, **214**, 1366.

BOND, H. E. (1962) A Sex-associated Protein in Liver Tissue of the Rat and its Response to Endocrine Manipulation. *Nature, Lond.*, **196**, 242.

CHEN, K. F. (1967) Removal of Fatty Acids from Serum Albumin by Charcoal Treatment. *J. biol. Chem.*, **242**, 173.

CHORDI, A., LLEDIAS, T., SANTAMARIA, P., ALVAREZ-MORENO, C. & ORTIZ DE LANDAZURIE (1969) Antigens of Human Liver Cells: Cytoplasmic, Mitochondrial and Microsomal Fractions. *Pathol. Eur.*, **4**, 209.

CLAUSEN, J., RASK-NIELSEN, R., CHRISTENSEN, H. E. & MUNKER, T. (1960) Two Transplantable Mouse Hepatomas Associated with an Increase of Metal-combining-globulin (Transferrin in Serum). *Cancer Res.*, **20**, 178.

CROWLE, A. J. (1961) *Immunodiffusion.* New York: Academic Press.

DECKERS, C. (1964) *Structure Antigénique de Tumeurs Expérimentales.* Bruxelles: Editions Arscia.

DUFOUR, D., BRASSARD, A., TREMBLAY, A. & LEMIEUX, S. (1967) Une Protéine Associée à la Croissance, à la Régénération Hépatique et au Développement Tumoral. *Path. Biol., Paris*, **15**, 757.

FRITZ, W. (1966) Vergleichende Untersuchungen löslicher Proteine aus Embryonalleber, Normalleber erwachsener Tiere und Transplantations-Tumoren der Ratte mit Hilfe der Agar und Papierelektrophorese. *Z. Naturf.*, **21B**, 482.

GRABAR, P. & BURTIN, P. (1964) *Immuno-electrophoretic Analysis.* Amsterdam: Elsevier.

HASE, T. & MAHIN, D. T. (1965) Electrophoretic and Immuno-electrophoretic Characterization of Soluble Cellular Proteins in Rat Liver Cell Sap. *J. Immun.*, **94**, 191.

HEREMANS, J., CLAUSEN, J., HEREMANS, M. T. & RASK-NIELSEN, K. (1959) Immunoelectrophoretic Characteristics of Normal Mouse Serums as a Basis for Studying Pathological Changes in Serums of Mice Carrying Transplantable Malignant Growths. *J. natn. Cancer Inst.*, **22**, 45.

KASHKIN, K. P. (1966) Immunochemical Analysis of the Proteins of Liver Cell Hyaloplasm and Cytoplasm Granules. *Folia biol., Praha*, **12**, 382.

KHRAMKOVA, N. I. & GUELSTEIN, V. I. (1965) Antigenic Structure of Mouse Hepatomas. V. Organospecific Liver Antigens and Embryonic-Globulin in Hepatomas of Mice Induced with Orthoaminoazotoluene (AAT). *Neoplasma*, **12**, 239.

KITAGAWA, M., TANIGAKI, N., YAGI, Y., PLANINSEK, J. & PRESSMAN, D. (1966) Carcinogen-binding Antigens in Rat Liver Microsomes. *Cancer Res.*, **26**, 752.

LEBOUTON, A. V. (1967) Electrophoretic Separation of Soluble Rat Liver Cell Proteins on Gelatinized Cellulose Acetate. *Anal. Biochem.*, **20**, 550.

LOUIS, C. J. & BLUNCK, J. M. (1970) The Isolation of Normal Rat Liver h-proteins and the Immunological Reactions of Mouse Anti-rat Liver h-protein. *Cancer Res.*, **30**, 2043.

LUNDKVIST, U. & PERLMANN, P. (1966) Immunochemical Studies of Submicrosomal Membranes from Liver of Normal and Phenobarbital-treated Rats. *Science, N.Y.*, **152**, 780.

NIKOLAEV, A. I., LI, M. I. & MIL'MON, M.SH. (1967) Immunological Processes in Animals after Administration of Cancerogenic Agents and Antibiotics. *Byull. eksp. Biol. Med.*, **63**, 93.

ROSSOWKI, W., WEINRAUDER, H. & HIEROWSKI, M. (1963) Immunochemical Studies on Cellular Fractions of the Differentiating Rat Liver. *Bull. Acad. pol. Sci. Sér. Sci. Biol.*, **11**, 579.

SALERNO, A., COURCON, J. & GRABAR, P. (1967) Analyse Immunochimique des Constituants Solubles du Thymus de Rat Normal. *Annls Inst. Pasteur, Paris*, **112**, 38.

SCHEIDEGGER, J. J. (1955) Une Micro-méthode de l'Immunoélectrophorèse. *Int. Archs Allergy appl. Immun.*, **7**, 103.

SCHWENKE, K. D. & KUJAWA, M. (1967) Elektrophoretische Untersuchungen der Serum- und löslichen Leberproteine von Ratten mit Transplantations-tumoren und normalen Ratten. *Z. Krebsforsch.*, **70**, 64.

SOROF, S., YOUNG, E. M., McCUE, M. M. & FETTERMAN, P. L. (1963) Zonal Electrophoresis of the Soluble Proteins of Liver and Tumor in Azo-dye Carcinogenesis. *Cancer Res.*, **23**, 864.

STANISLAWKLI, M., OIGSOLD, S., URIEL, J. & AVRAMEAS, S. (1964) Étude Immunochimique des Antigènes du Foie de Rat. In *Protides of the Biological Fluids.* Ed. H. Peeters. Amsterdam: Elsevier. p. 228.

SUNTZEFF, V. & DAVENPORT, G. R. (1965) The Protein Profiles of Primary Tumors and their Lung Metastases. *Cancer Res.*, **25**, 896.

SZFARARZ, D., YAMAMOTO, R. S. & WEISBURGER, J. H. (1969) Hormonal Control of Serum Albumin Synthesis (34080). *Proc. Soc. exp. Biol. Med.*, **131**, 1250.

TAKAYANAGI, N. (1966) Immunological Analysis of Extracts from Livers in the Course of Carcinogenesis. *Gann*, **57**, 577.

WEISBURGER, J. H., PAI, S. R., YAMAMOTO, R. S., KORZIS, J. & WEISBURGER, E. K. (1968) Pituitary Hormones and the Mechanism of Induction of Liver Cancer with N-hydroxy-N-2-fluorenylacetamide. *Israel J. med. Sci.*, **4**, 1223.

WIEME, R. J. (1965) *Agar Gel Electrophoresis.* Amsterdam: Elsevier.

YAMAMOTO, R. S., GLASS, R. M., FRANKEL, H. M. & WEISBURGER, J. H. (1968) Inhibition of the Toxicity and Carcinogenicity of N-2-fluorenylacetamide by Acetanilide. *Toxic. appl. Pharmac,.* **13**, 108.